# Angioregulatory microRNAs in breast cancer*:* Molecular mechanistic basis and implications for therapeutic strategies

**DOI:** 10.1016/j.jare.2021.06.019

**Published:** 2021-06-26

**Authors:** Mohammad Hasan Soheilifar, Nastaran Masoudi-Khoram, Soheil Madadi, Sima Nobari, Hamid Maadi, Hoda Keshmiri Neghab, Razieh Amini, Mahboubeh Pishnamazi

**Affiliations:** a Department of Medical Laser, Medical Laser Research Center, Yara Institute, ACECR, Tehran, Iran; b Research Center for Molecular Medicine, School of Medicine, Hamadan University of Medical Sciences, Hamadan, Iran; c Department of Biophysics, Faculty of Biological Sciences, Tarbiat Modares University, Tehran, Iran; d Department of Pharmaceutical Biotechnology, School of Pharmacy, Hamadan University of Medical Sciences, Hamadan, Iran; e Department of Oncology, Cross Cancer Institute, University of Alberta, Edmonton, Alberta, Canada; f Department of Photo Healing and Regeneration, Medical Laser Research Center, Yara Institute, ACECR, Tehran, Iran; g Department of Chemical Sciences, Bernal Institute, University of Limerick, Limerick, Ireland

**Keywords:** Breast cancer, Angioregulatory microRNA, Angiogenesis, Vascular mimicry, Anti-angiogenic therapeutics

## Abstract

**Background:**

Cancer-associated angiogenesis is a fundamental process in tumor growth and metastasis. A variety of signaling regulators and pathways contribute to establish neovascularization, among them as small endogenous non-coding RNAs, microRNAs (miRNAs) play prominent dual regulatory function in breast cancer (BC) angiogenesis.

**Aim of Review:**

This review aims at describing the current state-of-the-art in BC angiogenesis-mediated by angioregulatory miRNAs, and an overview of miRNAs dysregulation association with the anti-angiogenic response in addition to potential clinical application of miRNAs-based therapeutics.

**Key Scientific Concepts of Review:**

Angioregulatory miRNA–target gene interaction is not only involved in sprouting vessels of breast tumors but also, trans-differentiation of BC cells to endothelial cells (ECs) in a process termed vasculogenic mimicry. Using canonical and non-canonical angiogenesis pathways, the tumor cell employs the oncogenic characteristics such as miRNAs dysregulation to increase survival, proliferation, oxygen and nutrient supply, and treatment resistance. Angioregulatory miRNAs in BC cells and their microenvironment have therapeutic potential in cancer treatment. Although, miRNAs dysregulation can serve as tumor biomarker nevertheless, due to the association of miRNAs dysregulation with anti-angiogenic resistant phenotype, clinical benefits of anti-angiogenic therapy might be challenging in BC. Hence, unveiling the molecular mechanism underlying angioregulatory miRNAs sparked a booming interest in finding new treatment strategies such as miRNA-based therapies in BC.

## Highlights

• Cancer-associated angiogenesis is a fundamental process in tumor growth and metastasis.
• Angioregulatory miRNA–target gene interaction is not only involved in sprouting vessels of breast tumors but also, trans-differentiation of breast cancer cells to endothelial cells in a process termed vasculogenic mimicry.
• Successful targeting of tumor angiogenesis is still a missing link in the treatment of Breast cancer (BC) due to the low effectiveness of anti-angiogenic therapies in this cancer.
• Response to anti-angiogenic therapeutics are controlled by a miRNAs, so the identification of interaction networks of miRNAs–targets can be applicable in determining anti-angiogeneic therapy and new biomarkers in BC.
• Angioregulatory miRNAs in breast cancer cells and their microenvironment have therapeutic potential in cancer treatment.

## Graphical abstract

**Figure:**
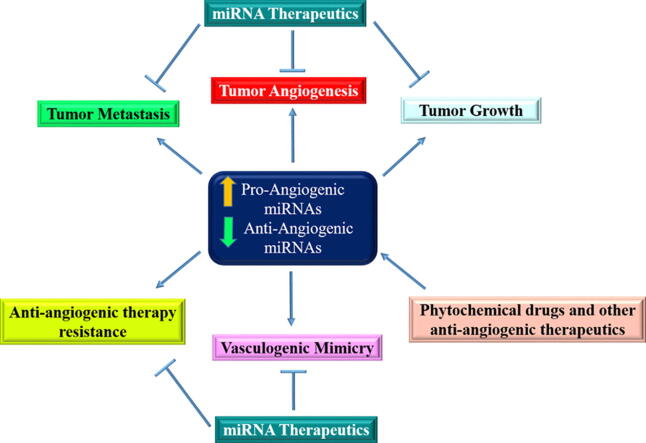

## Introduction

**Fig. 1:**
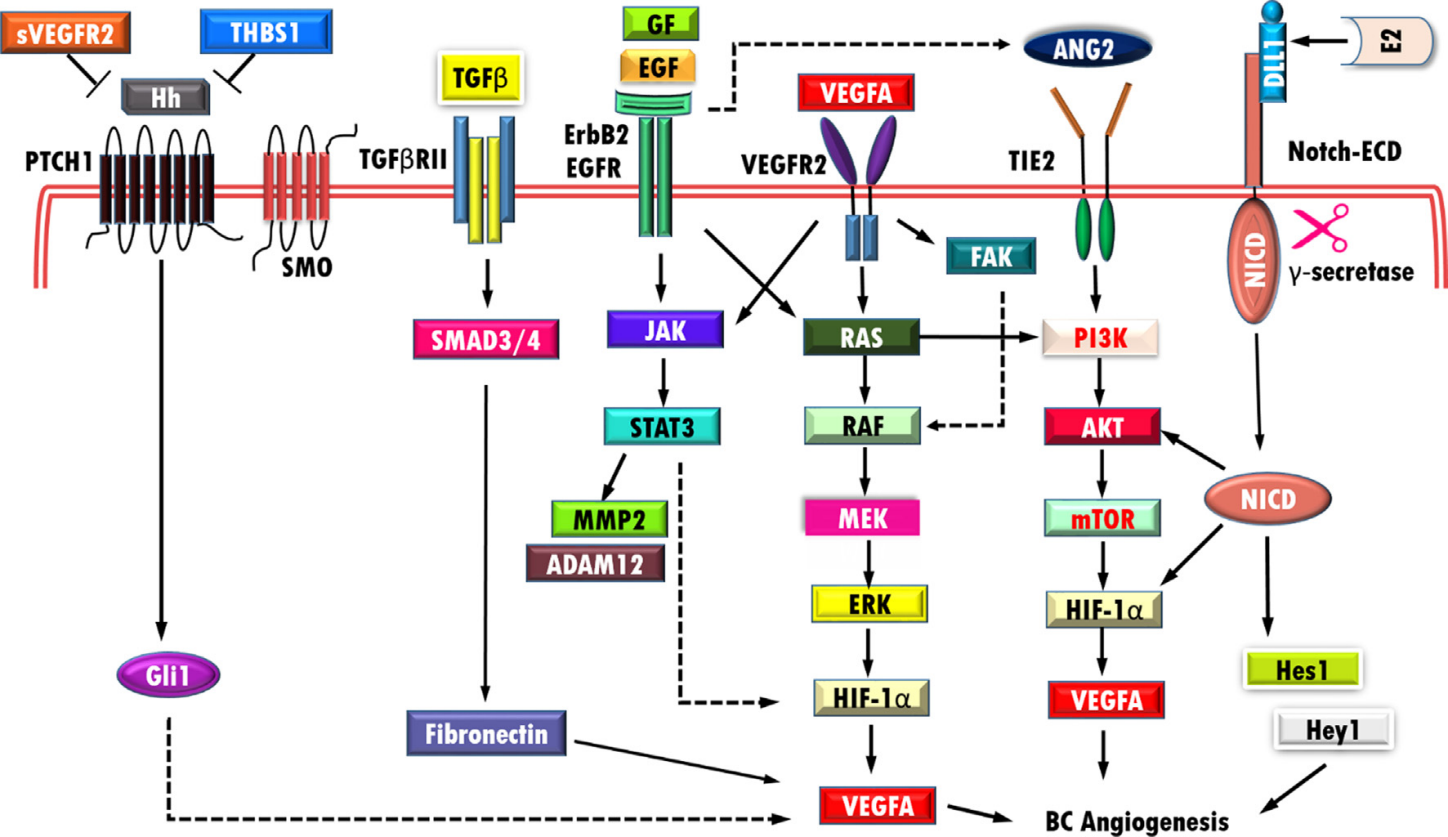
Schematic representation of molecular mediators in BC angiogenesis. PI3K/AKT/mTOR/VEGF, MAPK, STAT3, and Notch are major angiogenesis pathways in BC. VEGFA is a key angiogenic factor that involves in BC angiogenesis. Soluble VEGF receptor-2 (sVEGFR-2), vascular endothelial growth factor (VEGF), thrombospondin-1 (TSP-1), Glioma-associated oncogene homolog1 protein (Gli1), Hedgehog (Hh), Growth factor (GF) epidermal growth factor (EGF), Angiopoietin-2 (Ang-2), extracellular signal-regulated kinases (ERK), mitogen-activated protein/extracellular signal-regulated kinase kinase (MEK), Phosphoinositide 3**-**kinase (PI3K), Transforming growth factor-β (TGF-β), Estradiol (E2), intracellular domain of the notch protein (NICD), Extracellular domain (ECD), Janus kinase (JAK), signal transducer and activator of transcription (STAT), focal adhesion (FAK), PTCH1 (Patched 1), SMO (Smoothened).

Breast cancer (BC) is one of the most common type of malignancy in females worldwide. The major frequent cause of BC mortality is metastasis [1] which is predominantly ascribe to angiogenesis. Angiogenesis is a process by which new blood vessels form to supply oxygen and nutrients within tumors. Tumor cells are able to produce and secrete the various chemical factors including vascular endothelial growth factor (VEGF), fibroblast growth factor (FGF), platelet-derived growth factor (PDGF), angiopoietins (Ang), hypoxia-inducible factor (HIF), insulin-like growth factor (IGF), transforming growth factor-beta (TGFβ), matrix metalloproteinase (MMP), and tumor necrosis factor (TNF) into tumor microenvironments [2]. The angiogenic process is regulated through imbalance between pro-angiogenic and anti- angiogenic signals [3]. Moreover, the hypoxic condition and response to some anti-tumoral therapies will lead to angiogenic switch and tumor neovascularization, thereby maintaining the progression of the tumor. Higher circulating angiogenesis-related markers in post-mastectomy BC patients compared to control highlighted the significance of angiogenesis in tumor progression in BC patinets even after surgery [4]. Benign and pre-malignant mammary tumors with increased microvessel density (MVD) consist of a higher probability of metastatic spread [5]. Accordingly, understanding the mechanisms underlying the regulation of angiogenesis is critical in BC therapy (see Fig. 1).

The angiogenic switch could be induced by genetic and epigenetic alterations [6], [7]. One of the dominant players in epigenetic orchestration of angiogenesis is microRNAs (miRNAs). MiRNAs are endogenous ~22 nt non-coding RNAs regulate gene expression through translation inhibition or messenger RNAs (mRNA)s degradation. Target sites of miRNAs are in 3′ untranslated region (UTR), 5′ UTR or protein coding sequence of mRNAs. Moreover, miRNAs can upregulate target genes through activation of mRNA translation or repression of suppressive factors in a selective manner and distinct cellular circumstance [8]. Numerous cancer cells functions can be controlled by miRNAs, which confer new features to tumor cells including high proliferation rate, resistance to radiation and chemotherapy, invasiveness, metastasis, cancer stem cells (CSC)s maintenance as well as augmented angiogenesis in various types of cancers including BC [9], [10], [11], [12].

Clinical series, *in vitro*, and *in vivo* experiments analysis have revealed angioregulatory miRNAs can suppress or promote angiogenesis through hyperactivation or hypoactivation of tumor cell signaling mediators [13], [14]. The aggressive BC showed deregulated angioregulatory miRNAs and elevated pro-angiogenic factors that could mediate communication between tumor cell and its microenvironment [15], [16]. Phosphoinositide 3**-**kinase (PI3K)/AKT/mTOR/ vascular endothelial growth factor (VEGF), mitogen**-**activated protein kinase (MAPK), signal transducers and activators of transcription 3 (STAT3), and Notch are major signaling pathways that could be affected by angioregulatory miRNA in BC. PI3K/AKT and MAPK signaling pathways are usually hyper activated in various human malignancies. These pathways regulate various cellular processes including cell growth, apoptosis, proliferation, migration, and survival [17], [18]. Moreover, dysregulation of other signaling pathways such as STAT3, Notch, and Hedgehog (Hh) contributed in BC stem cell maintenance, metastasis, and chemoresistance [19], [20], [21].

The anti-angiogenic resistant phenotype may confer by angioregulatory miRNAs through downregulation of endogenous angiostatic factors like thrombospondin-1 (TSP-1), or upregulation of proangiogenic proteins like VEGF, moreover, activation of alternative angiogenesis pathways such as vasculogenic mimicry (VM) is another possible anti-angiogenic resistance mechanisms, which could be mediated by angioregulatory miRNAs.

Although- anti-angiogenic strategies in BC patients might improve progression-free survival, so far, successful treatment response remains an important challenge in BC anti-angiogenic therapies. On the other hand, the expression of these miRNAs can be modulated by anti-angiogenic therapeutics. Hence, understanding the molecular mechanisms underlying BC angiogenesis can pave the way for designing more effective miRNA-based treatments and identifying novel biomarkers. The present review will summarize the all findings toward the understanding mechanism of miRNA in regulation of angiogenesis, specifically in BC. Then we will discuss the utility of miRNAs as the potential therapeutic target in clinical investigation.

## Molecular players in BC angiogenesis: At a glance

Angiogenesis is the critical step in BC progresion and metastasis [22]. Several studies have been conducted to identify the molecular players of angiogenesis [23], [24], [25]. Among all factors, VEGF is the main regulator of angiogeneis. The VEGF family consists of five members including VEGFA, VEGFB, VEGFC, VEGFD, and VEGFE, which can bind to three VEGF receptors (VEGFR1, R2 and R3). VEGFA and VEGFB are responsible for angiogenesis, while VEGFC and VEGFD are involved in lymphangiogenesis [22], [26]. VGEFA binds to VEGFR2 which in turn activates mitogen-activated protein kinase (MAPK) signaling and phosphorylate ERK, followed by induction of HIF1α [27], [28].

Angiogenesis is also regulated by growth factor like TGF-β [29]. TGF-β1, one of the TGFβ isoform, has been recognized to has both pro-angiogenic and anti-angiogenic properties. TGF-β1 has been shown to stimulate Smad dependent angiogenesis. Smad3 has pro-angiogenic function by promoting VEGFA expression, while Smad2 induces TSP-1 production and inhibits angiogenesis [30]. In BC, TGF-β /SMAD/fibronectin axis activation during crosstalk between BC cells andcancer-associated fibroblasts (CAF)s can promote neovascularization by elevating pericyte-endothelium association [31].

Angiopoietins are the endothelial growth factors which act as the ligands for tyrosin kinase receptors (TIE1 and TIE2) in angiopoietins/TIE receptor pathways [32]. One of the angiopoietin family members, Ang-2 is highly expressed in many tumor cells and is correlated with poor prognosis of BC [33]. Ang-2 and VEGF expression significatly induced brain angiogenesis in BC models [34]. Ang-2/TIE2/VEGF axis invovled in BC cells co-cultured with HUVECs, sporadic and familial BC [35]. ErbB-2 leads to Ang-2 upregulation and activation of PI3K/AKT and MAPK pathways in BC, followed by HIF1α and VEGF activation [36], [37].

MMPs are a family of proteolytic enzymes, which are able to degrade extracellular matrix (ECM). Up to now, 23 MMPs have been recognized in human and classified into different groups (collagenases, gelatinases, stromelysins, matrilysins, transmembrane type I, transmembrane type II, glycosylphosphatidylinositol-anchored (GPI-anchored) MMPs and other MMPs) based on their structure and substrates [38]. MMPs can have different role in tumor progreassion including survival, angiogenesis, and metastasis. Moreover, many MMP proteins are khown to be upregulated in various tumors as well as BC. The overexpreesion of MMP1–3, MMP7–9, MMP11, and MMP13–15 have been reported in samples from BC patients [39]. MMP-2 and MMP-9 have been reported to regulate angiogenseis through induction of pro-angiogenic factors including VEGF, basic fibroblast growth factors (bFGF), TGF-β, and angiogenin [40]. In breast cancer, disintegrin and metalloproteinase domain-containing protein 12 (ADAM12) is one of the members of MMP family, which its overexpression could increase angiogenesis through epidermal growth factor receptor (EGFR)/STAT3/AKT in BC [41].

EGF and FGF also exhibited angioregulatory function through tyrosin kinase receptors. FGFR1 is the transmembrane tyrosin kinase receptor which can bind FGF-2 and activate JAK/ STAT3 pathway [42].

The overexpression of BP1 (isoform of DLX4) induced VEGF expression and activated PI3K/AKT pathway in estrogen receptor (ER) negative BC [43].

The Notch signaling pathway is essential for regulation of angiogenesis in tumor cells. This family of proteins comprise Notch receptors and their ligands (Jagged 1–2, Delta-like1, 3, 4). In response to activation of Notch pathway, the Notch intracellular domain (NICD) is relaesd and translocates to nucleus and induces the expression of Hairy/Enhancer of Split (HES) and HES-related genes [44]. Notch pathway seems to interact with angiogenic pathway through VEGF/VEGFR-2, TGF-β signaling pathway, EGFR, and PI3K signaling pathway [45]. Recently, DLL1 has been found to promote- angiogenesis in ERα positive luminal breast cancer [46].

Sonic hedgehog (Shh) signaling is another angiogenesis related pathway, which is dysregulated in various tumors, especially BC. This pathway initiates by binding Shh to a transmembrane receptor Patched 1 (PTCH 1) and release of smoothened (SMO), and thereby activation of glioma-associated oncogene homolog (GLI). GLI proteins (GLI1, GLI2 and GLI3) are the transcription factors can regulate the expression of multiple genes including ANG1/2, VEGF and Platelet-derived growth factor (PDGF) [47], [48]. It has been demonstrated that GLI1 and SMO expression are higher in TNBC cells, and correlated with VEGFR2 expression. The media of TNBC cells containing NVP-LDE225 (SMO antagonist) and Bevacizumab (a monoclonal antibody against VEGF) reduced angiogenesis of HUVECs [49].

## Pro-angiogenic and anti-angiogenic miRNAs in BC

**Fig. 2:**
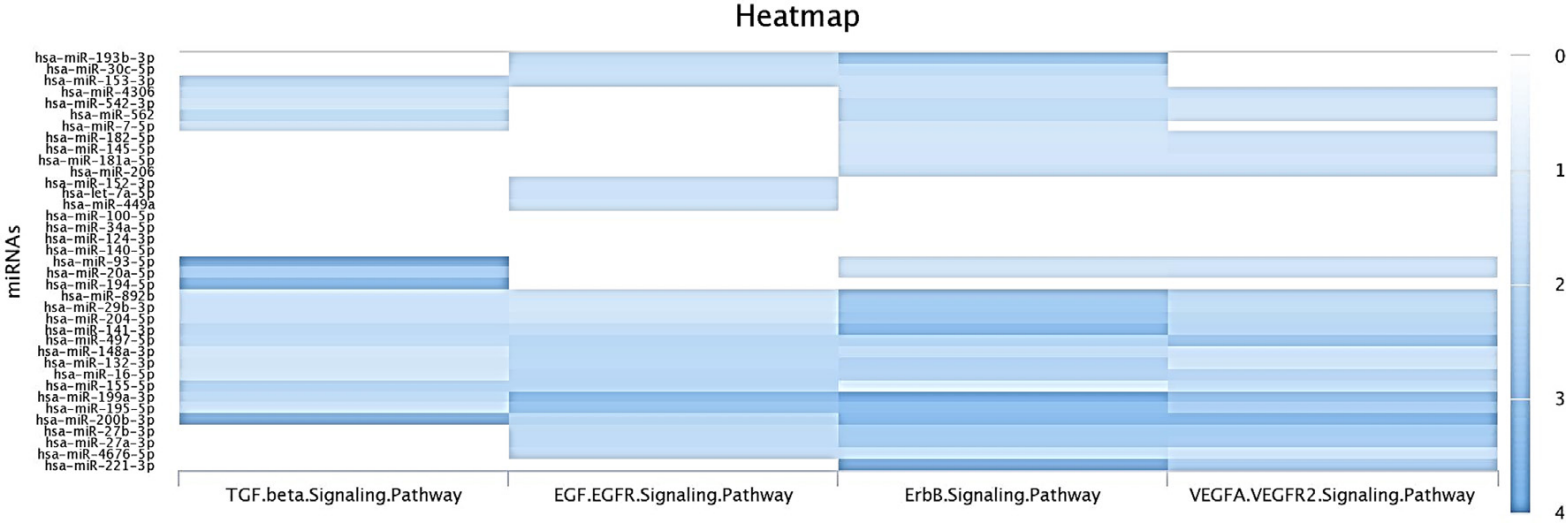
In silico prediction of major signaling pathways asscotiated with angioregulatory miRNAs in BC by miRPathDB v2.0. The results indicated that the majority of these miRNAs are invovled in ErbB (HER) and VEGFA/VEGFR2 signaling pathways. Hence, understanding VEGFA expression regulation by miRNAs would be valuble in unraveling precise mechanisms underlying BC angiogenesis.

**Fig. 3:**
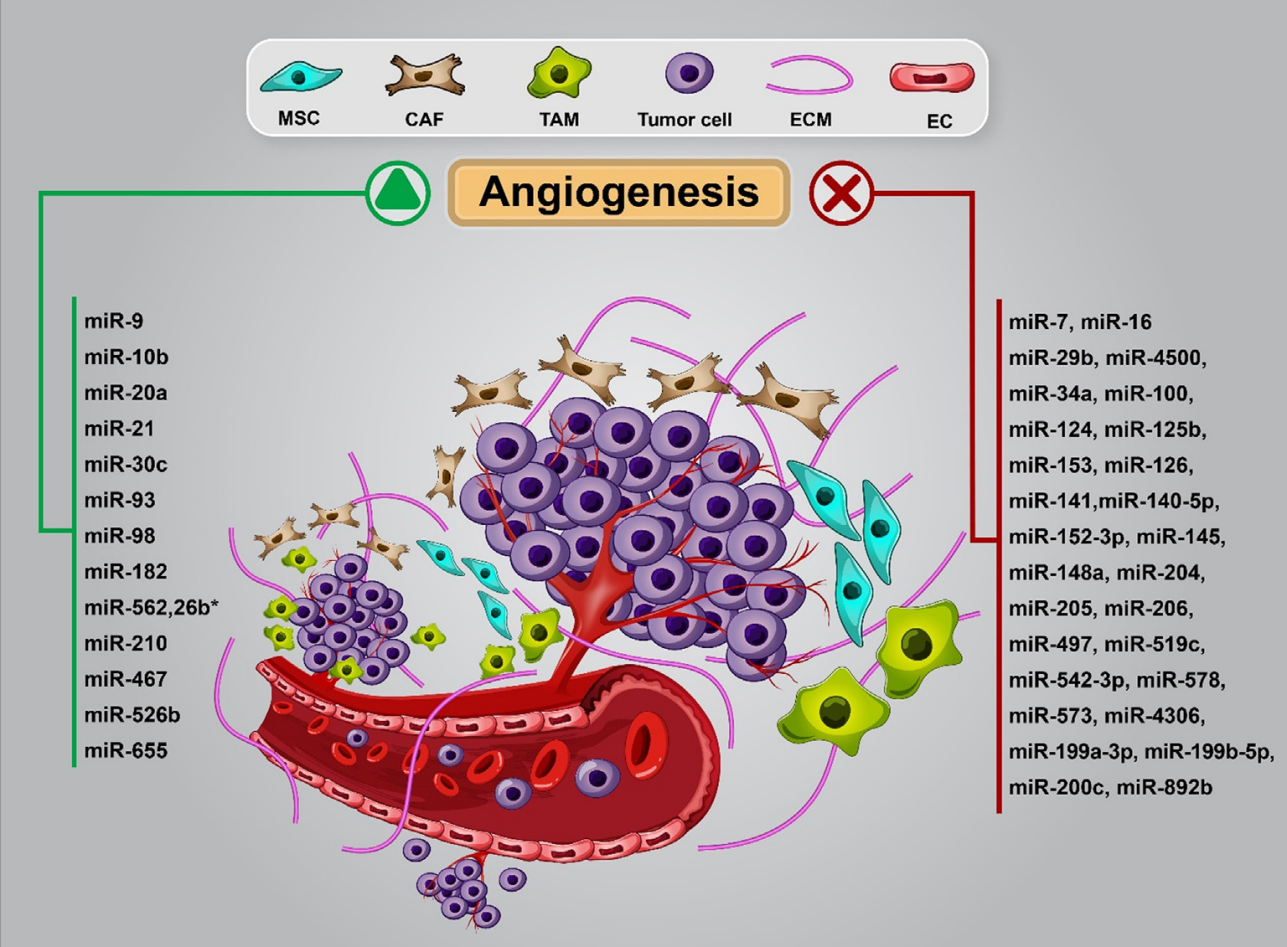
Anti-angiogenic and pro-angiogenic miRNAs in BC cells and tumor microenvironment. Angioregulatory miRNAs by modulating of angiogenesis-related targets could suppress or promote BC angiogenesis. Angiogenesis is a crucial step in metastasis and can facilitate tumor metastasis. Cancer cells and TME communication involved in BC angiogenesis. For instance, exosomal miRNAs such as miR-542-3p, miR-100 and miR-205 may regulate BC angiogenesis through macrophages, MSCs and CAFs respectively. MSC, mesenchymal stem cell; CAF, cancer-associated fibroblasts; TAM, tumor-associated macrophages; ECM, extracellular matrix; EC, endothelial cell.

MiRNAs exacerbate or suppress angiogenic potential of tumor cells, and could be incorporate into nano-sized vesicular structures and affect tumor microenviorenment (TME). By investigation of experimentally validated interactions of BC angioregulatory miRNAs in DIANA-miRPath v3. 0 web-server [50] we identified that focal adhesion, MAPK, HIF-1α, PI3K/AKT, mTOR, TGF-β, and VEGF signaling pathways are involved in miRNAs-mediated BC angiogenesis. Moreover, by predicting interactions of BC angioregulatory miRNAs in miRPathDB v2.0 [51] we distinguished that TGF-β, EGFR, ErbB and VEGF are the prominent possible pathways underlying most of these miRNAs in angiogenesis (Fig. 2). The angiogenic or anti-angiogenic role of these miRNAs in other types of cancer (excluding BC) summarized in Table 1. Angioregulatory miRNAs can regulate tumor vasculature by fine-tune modulating signaling pathways in tumor cells and TME components including ECs, cancer-associated fibroblasts (CAFs), tumor-associated macrophages (TAMs), and mesenchymal stem cells (MSCs) (Fig. 3). ECs-BC cells reciprocal crosstalk mediated by miRNAs is the most well-studied communication in BC angiogenesis and metastasis. BC cells co-culture with ECs induce pro-angiogenic response in tumor cells [52]. ECs Jagged1 promotes metastatic and stemness capabilities of BC cells through Notch signaling [53]. Therefore, besides the major role of ECs in angiogenesis, other tumorigenic processes can be regulated by ECs and-BC cells crosstalk. Exosomal angioregulatory miRNAs shuttle between tumor cells and TME cells or vice versa, reprogramme target cells to facilitate or impede angiogenesis. In the following, crosstalk between BC-related miRNAs and their targets in different signaling pathways is discussed.

**Table 1:** BC-related angioregulatory miRNAs in other types of cancer.

miRNAs	Tumor type	Anti/pro- angiogenic or both	Target genes and/or molecular pathways	Ref.
miR-7	Glioblastoma	Anti	OGT	[187]
miR-16	Multiple myeloma	Anti	VEGFA	[188]
miR-29b	Endometrial carcinoma	Anti	VEGF, AKT, mTOR, ERK nd BCL-2	[189]
	Hepatocellular carcinoma	Anti	MMP-2	[190]
	Cervical cancer	Anti	STAT3	[191]
miR-34a	Pancreatic ductal adenocarcinoma	Anti	Nocth1	[192]
miR-93-5p	Astrocytoma	Anti	integrin-β8	[193]
	Neuroblastoma	Anti	IL-8 , VEGF	[194]
miR-98	Hepatocellular carcinoma	Anti	Ang-1, FGF-1	[195]
miR-124-3p	Glioblastoma multiforme	Anti	NRP-1 PI3K/Akt/NF-κB	[196]
miR-126	Hepatocellular carcinoma	Anti	EGFL7 ERK signaling pathway	[197]
	Gastric cancer	Anti	MAPK/ERK Akt/mTOR VEGF expression	[198]
	Cervical cancer	Anti	ADM	[199]
miR-132	lung cancer	Anti	–	[200]
miR-140-5p	Laryngeal cancer	Anti	VEGFA	[201]
miR-141	Pancreatic cancer	Anti	TM4SF1 VEGFA E cadherin	[202]
miR-145	Colon cancer	Anti	p70S6K1	[203]
	Neuroblastoma	Anti	HIF-2α	[204]
	Pancreatic cancer	Anti	Angiopoietin-2	[205]
	Thyroid cancer	Anti	VEGF, HIF1α	[206]
	Ovarian cancer	Anti	MTDH	[207]
miR-152-3p	Gastric cancer	Anti	–	[208]
miR-153	Bladder cancer	Anti	IDO1 STAT3/VEGF signaling pathway	[209]
miR-206	NSCLC	Anti	14–3-3 zeta STAT3/HIF-1α/VEGF pathway	[210]
			c-Met PI3k/Akt/mTOR signaling pathway	[211]
	Pancreatic cancer	Anti	VEGFC	[212]
	Colorectal cancer	Anti	Met/ERK/Elk-1/HIF-1α/ VEGF-A signaling pathway	[213]
miR-449a	Prostate cancer	Anti	PrLZ	[214]
miR-497	NSCLC	Anti	HDGF	[215]
	Ovarian cancer	Anti	VEGFA VEGFR2-mediated PI3K/AKT, MAPK/ERK pathways	[216]
	Hepatocellular carcinoma	Anti	VEGFA , AEG-1	[217]
	Glioblastoma	Anti	VEGF	[218]
miR-519c	Lung adenocarcinoma	Anti	HIF-1α	[121]
miR-10b	Glioblastoma	Pro	VEGF, IL8, TGFB2	[219]
miR-20a	Colon cancer	Pro	ZBTB10, ZBTB4 Specificity protein (SP)	[220], [221]
miR-21	Astrocytoma	Pro	HIF-1α, VEGF	[222]
	Prostate cancer	Pro	PTEN AKT, ERK1/2 pathway HIF-1α ,VEGF	[223]
miR-27a	Colon cancer	Pro	SAMD4	[224]
miR-148a	Glioblastoma	Pro	QKI	[225]
	Glioma	Pro	ERRFI1 EGFR/MAPK signaling pathway	[226]
miR-155	Gastric Cancer	Pro	c-MYB, VEGF	[227]
miR-181a-5p	Colorectal cancer	Pro	SRCIN1, VEGF	[228]
miR-194	Colon cancer	Pro	TSP-1 P53- mediated pathway	[229]
miR-210	Hepatocellular carcinoma	Pro	FGFRL1 SMAD4 , STAT6	[230], [231]
	Lung cancer	Pro	VEGFA ,MMP9, FGF2 JAK2/STAT3 signaling pathway TIMP-1 PI3K/Akt pathway	[232], [233]
miR-9	Glioma	Both	PHD3 HIF-1α/VEGF signaling pathway	[234]
	Nasopharyngeal carcinoma	Both	MDH PDK1/AKT signaling pathway	[235]
	Cervical cancer and Melanoma	Both	SOCS5 JAK/STAT signaling pathway	[236], [237]
miR-182	Colon cancer	Both	VEGF-C, VEGF-A, VEGFR-2, VEGFR-3 ERK and AKT phosphorylation	[238]
	Glioblastoma	Both	KLF2 KLF4	[239]
	Hepatocellular carcinoma	Both	RASA1	[240]
miR-200	Pancreatic cancer	Both	–	[241]
	NSCLC	Both	VEGFR2	[242]
	Prostate Cancer	Both	–	[243]
	Chondrosarcoma	Both	Chemokine CCL5 PI3K/Akt signaling pathway	[244]
	Bladder cancer	Both	HIF-1α, VEGF Akt2/mTOR pathway	[245]
miR-204	Ovarian cancer	Both	TSP-1	[246]
	Head and neck squamous cell carcinoma	Both	JAK2-STAT1 pathway	[247]
miR-205	NSCLC	Both	PTEN, PHLPP2 AKT pathway	[248]
	Ovarian cancer	Both	PTEN AKT signaling activation	[249], [250]
	Thyroid cancer	Both	ZEB1 VEGFA	[251]

ADM: Adrenomedullin; AEG-1astrocyte elevated gene-1; EGFL7: epidermal growth factor-like domain 7; ERRFI1: ERBB receptor feedback inhibitor 1; IDO1: indoleamine 2,3-dioxygenase 1; NRP-1:neurophilin neurophilin; MTDH: metadherin; HDGF: Heparin Binding Growth Factor; OGT: O-linked β-N-acetyl glucosamine transferase; PHD3: Prolyl hydroxylase-3; PHLPP2: PH Domain And Leucine Rich Repeat Protein Phosphatase 2; PrLZ: prostate leucine zipper; QKI: Quaking; RASA1: Ras p21 Protein Activator 1; SOCS5: suppressor of cytokine signaling 5; SRCIN1: SRC kinase signaling inhibitor 1; TIMP-1: Tissue inhibitor of metalloproteinases-1; TM4SF1: transmembrane-4-L-six-family-1; TSP-1: Thrombospondin 1; ZBTB10: Zinc Finger And BTB Domain Containing 10; ZBTB4: Zinc Finger And BTB Domain Containing 4.

### Anti-angiogenic miRNAs in BC

MiR-7: MiR-7 directly targets EGFR and Raf1 in BC [54]. EGFR activation can regulate the secretion of several angiogenic regulating factors, such as bFGF, VEGF and interleukin-8 (IL-8) [55]. Anti-angiogenic effect of miR-7 is mediated by targeting chemokine receptors CXCR4 and CXCR7 in BC, which are receptors for the stromal cell-derived factor-1 alpha (SDF-1*α*)*/* CXCL12 chemokines [56]. On the other hand, SDF-1*α* can promote tumor angiogenesis indirectly through enhancing the secretion of VEGF and IL-8 from tumor cells [57]. Therefore, miR-7 could affect BC angiogenesis through modulation of TME.

MiR-16: Lee *et al.* found that miR-16 shuttled by MSC-derived exosomes reduced the expression of VEGF in the murine BC cells which was also confirmed by using miR-16 inhibitor. They further established the inhibitory role of miR-16 on the angiogenesis which could significantly decrease the rate of proliferation and migration of the murine ECs. Also, tumor growth, tumor weight and the mRNA level of VEGF were significantly reduced in the BALB/c mice injected with MSC-derived exosomes [58]. Hence, exosomal miR-16 could modulate angiogenesis in the BC through intercellular communications.

MiR-29b: MiR-29b that belongs to miR-29 family, was shown as the tumor suppressor miRNAs in various type of cancer [59]. In BC, miR-29b has a dual role in either promoting cell migration and invasion [60] or inhibiting metastasis [61]. GATA3 (GATA-binding protein 3) is a zinc-finger transcription factor which is critical for differentiation of mammary epithelial cells, and its mutation involved in poor prognosis of BC [62]. Jonathan *et al.* showed that GATA3 repressed migration and regulated TME by inducing miR-29b. They demonstrated that the absence of miR-29b, even in GATA3 expressing cells, impaired differentiation and promoted metastasis. In addition, miR-29b significantly reduced the expression of pro-angiogenic proteins like MMP-9 and VEGFA [61]. A similar study has also shown that miR-29b is able to inhibit angiogenesis by targeting Akt3 expression, and subsequently reduced the expression of VEGF. Moreover, miR-29b is shown to suppress angiogenesis and tumor growth *in vivo* [63].

MiR-30c: McCann *et al*. have shown that downregulation of miR-30c by TGF-β in BC could trigger fibrin formation and then through repression of serpin-1 promoted angiogenesis in the distinct phenotype of tumor endothelial cells (TEC) [64]. Fibrin formation could play as a scaffold and protein carrier for ECs spreading and survival which supports tumor neo-angiogenesis [65]. Due to TEC heterogeneous phenotype TGF-β /miR-30c/serpin-1 signified the precision medicine approach in cancer therapy.

MiR-34a: MiR-34a is a known anti-metastatic miRNA located at human chromosome 1p36.22 region [66]. MiR-34a is directly linked to Notch1 expression in ECs, and is associated with tumor angiogenesis in TNBC [67]. MiR-34a directly targets 3′-UTR of eukaryotic elongation factor 2 kinase (eEF2K) and forkhead box M1 (FOXM1) mRNA gene, leading to inhibition of cell invasion and angiogenesis in TNBC [68]. FOXM1 is overexpressed in BC, and associated with angiogenesis in TNBC [69].

MiR-98: Anti-angiogenic role of miR-98 has been approved in Siragam *et al*. study [70]. They have shown that miR-98 overexpression in ECs induced tube formation by targeting activin receptor-like kinase (ALK4), and MMP11. Moreover, co-culture of ECs with miR-98 or anti-miR-98 transfected BC cells quenched and activated angiogenesis, respectively. This observation was also confirmed in BC mouse model.

MiR-100: MiR-100 can modulate BC angiogenesis through paracrine communication between cancer cells and MSCs. Pakravan and coauthors disclosed exosomal miR-100 delivery into BC cells suppressed mTOR/HIF-1α axis and decreased VEGF expression and secretion into conditioned-medium (CM) [71]. They further demonstrated HUVECs exposure to CM of BC cells treated with MSCs exosomes leads to impaired tube formation, proliferation, and migration which are associated with miR-100/mTOR/HIF-1α/VEGF signaling axis.

MiR-124: Jiang *et al.* identified miR-124 as the most significantly estrogen-suppressed miRNA in the estrogen receptor (ER) positive BC cells. The inhibition of miR-124 markedly increased cell growth, migration, and invasion of the BC cells, indicating the role of miR-124 in BC progression. AKT2, a well-known oncogene, was further identified as a direct target of miR-124 and the results showed a negative correlation between the expression levels of miR-124 and AKT2 in the human BC tissues. MiR-124-mediated AKT2 suppression led to a significant reduction in the tumor size, tumor weight and the number of microvessels, thus suppressed ER-positive BC angiogenesis *in vivo* [72].

MiR-125b: Ray *et al*. noted that over-expression of miR-125b reduced mRNA and protein levels of serum amyloid A-activating factor (SAF-1). As SAF-1 is a transcriptional inducer of VEGF [73] miR-125-mediated repression of SAF-1 led to the reduction in mRNA and protein levels of VEGF in the BC cells. Furthermore, increased levels of miR-125b lowered the migration rate of HUVECs and cellular invasive phenotypes of BC cells [74]. Zhou *et al*. observed that overexpression of miR-125b down-regulated ERBB2 expression that led to the decreased levels of VEGF in the heat-denatured HUVECs. They suggested that ERBB2 may be a mediator between miR-125b and VEGF. They also found that increased levels of miR-125b impaired the migration and tube formation ability of HUVECs [75].

MiR-126: The endothelial specific miR-126 located within intron 6 and 7 of the epidermal growth factor‐like‐domain 7 gene (EGFL7), plays controversial role in cancers [76]. MiR-126 plays anti-angiogenic role in BC through negative regulation of VEGFA/PI3K/ERK via direct targeting VEGFA and phosphoinositide-3-kinase regulatory subunit 2 (PI3KR2/p85-β) [77]. MiR-126/miR-126* directly inhibit the expression of SDF-1α in BC [78]. SDF-1α can promote angiogenesis in both primary and metastatic BC, through crosstalk with typical signaling pathways such as PI3K/AKT, NF-κB, STAT3, as well as stem-cell related pathways like Notch, Wnt, and SHH [79]. In addition, miR-126 indirectly suppresses the expression of chemokine (C–C motif) ligand 2 (Ccl2), which recruits inflammatory monocytes into the tumor stroma in SDF-1α-dependent manner to promote BC angiogenesis [78]. Various studies demonstrated that hypoxia-induced SDF-1α by recruitment of bone-marrow derived cells involved in anti-angiogenic therapy resistance [80]. Therefore, MiR-126/SDf-1α might be considered as predictive biomarker for anti-angiogenic treatment response in BC.

MiR-140-5p: Several studies have suggested tumor suppressive role for miR-140-5p in BC [81], [82], [83]. Analyzing the expression of miR-140-5p in normal and neoplastic breast tissues has revealed that miR-140-5p is significantly downregulated in breast tumors [81]. Of note, lower expression of miR-140-5p have been demonstrated in breast tumors at higher stages and with more invasive properties [81]. Similar results have been found by He *et al*. who reported the decreased expression level as well as increased promoter methylation of miR-140-5p in TCGA invasive breast tumor samples [82]. It has been shown that the CM from MCF-7 and MDA-MB-231 BCE cells transfected with miR-140-5p have lower ability to induce the tube formation in HUVEC cells in comparison with CM from untransfected cells [81]. Using the luciferase reporter assay, VEGFA is identified as a direct target of miR-140-5p [81]. In addition, *in vivo* experiments have indicated that VEGFA has lower expression in MCF-7 tumor xenografts when the tumor stably expressed miR-140-5p [81]. Therefore, the anti-angiogenic property of miR-140-5p is attributed to its ability to inhibit the expression of VEGFA as a pro-angiogenic growth factor in BC.

MiR-141: Kaban *et al.* measured the levels of miR-141 in the human TN-BC cells and found that miR-141 was down-regulated in the BC cells compared to the control cells. They also found that treatment with chitosan/miR‐141 nanoplexes significantly reduced the production of VEGF in these cells. However, chitosan/miR‐141 nanoplexes increased the expression levels of two metastatic factors, Tinagl1 and Igfbp‐4, in the BC cells and thus the role of miR-141 in the angiogenesis needs further investigation [84]. MiR-141 suppressed IL-8 and CXCL1 in basal-like BC which led to reduction in tumor growth and MVD [85].

MiR-145: Several studies have revealed anti-angiogenic role of miR-145 in cancers. Zou and coauthors showed miR-145 transfection into HUVECs and BC cells leads to decreased angiogenesis and invasion, respectively [86]. Moreover, MVD and tumor cell proliferation assessment in mouse models of BC exhibited that miR-145 overexpression resulted in decreased MVD and tumor growth by targeting VEGF, insulin receptor substrate (IRS), and N-RAS directly, as well as, important mediators in the PI3K/AKT signaling pathway such as AKT, mTOR, phosphatidylinositol-4,5-bisphosphate 3-Kinase catalytic subunit alpha (PIK3CA), and phosphoinositide-3-kinase regulatory subunit 1 (PIK3R1) indirectly [86].

MiR-152-3p and miR-148: The family of miR-148/152 consists of miR-148a, miR-148b, and miR-152 which miR-152 can form two mature miR-152-5p and-3p [87]. MiR-152-3p has been known as the tumor suppressor, which was downregulated in BC [88], [89], [90]. Xu *et al.* have demonstrated that upregulation of miR-152 significantly inhibits breast tumor angiogenesis [90]. It has been shown that overexpression of miR-152-3p in MDA-MB-468 BCE cells reduces the protein expression of insulin-like growth factor-I receptor (IGF-IR), HIF-1α, and VEGF [88]. Further *in vitro* studies in BC cells have suggested that miR-152 can attenuate the expression of HIF-1α and VEGF as well as the phosphorylation of AKT and extracellular signal-regulated kinases (ERK) through direct targeting of IGF-IR and insulin receptor substrate 1 (IRS1) transcripts [90]. In addition, β-catenin which plays a significant role in VEGFA expression is determined as another target of miR-152 in BC cells [89]. Collectively, these results provide evidence showing that miR-152-3p has an anti-angiogenic role in BC tumors. Yu *et al*. showed that the expression of miR-148a was significantly decreased in the human BC cells. They further identified ERBB3 and its downstream molecules, AKT and ERK, as direct targets of miR-148a. They also revealed that miR-148a inhibited tumor angiogenesis via targeting ERBB3. The levels of phosphorylated AKT and ERK were remarkably suppressed in the BC cells expressing miR-148a which reduced AKT and ERK activation. In addition, miR-148a overexpression inhibited HIF-1α expression *in vitro*. Forced expression of miR-148a, statistically inhibited cancer cell-inducing angiogenesis responses by 45% in the chicken chorioallantoic membrane of the chicken embryos [91].

MiR-153: MiR-153 was identified as an under expressed miRNA in BC cell lines and samples compared to normal cells and tissues. Low expression of miR-153 was associated with metastasis and poor prognosis of BC [92]. The expression of miR-153 has been found to promote apoptosis by targeting HECT domain E3 ubiquitin protein ligase 3 (HECTD3) [93] and inhibit proliferation and migration through suppressing runt-related transcription factor 2 (RUNX2) [92] and impaired zinc finger E-box-binding homeobox 2 (ZEB2)-mediated EMT [94] in BC cells. Liang *et al.* have investigated the effect of miR-153 on angiogenesis, and showed that it can target Ang1 [95]. Ang1 is the critical regulator of angiogenesis, which mediates vessels formation and induces migration [96]. The high expression of Ang1 was observed in BC samples. MiR-153 effectively reduced proliferation and migration of MCF7 cell line, and tube formation in HUVECs [95].

MiR-181a-5p: MiR-181a has been found to play different role in BC. Some data support the idea that miR-181a acts as a tumor suppressor miRNA, while others suggested that it can also promote metastasis and increase cell survival [97]. Li *et al.* showed that miR-181a-5p affect angiogenesis by targeting MMP14 in BC [50]. MMP14 is the one of transmembrane protease which can degrade extracellular matrix (ECM), leading to cancer cell migration. It also known to trigger pro-angiogenic signal through VEGF and TGFβ signaling pathway [40]. MMP14 is overexpressed in BC specimen, and it was negatively regulated by miR-181a-5p. The over expression of miR-181a-5p effectively reduced angiogenesis and invasion *in vivo* [50].

MiR-199a-3p: Huang *et al*. have reported GPER (G protein estrogen receptor) agonist treatment of TNBC cells leads to upregulation of miR-199a-3p expression, and CD151 downregulation [98]. Tetraspanin CD151 upregulated in BC vasculature, and by binding to laminin-binding integrins could regulate tumor angiogenesis [99]. It has been demonstrated miR-199a-3p could suppress epithelial-mesenchymal transition (EMT) and exhibit anti-angiogenic function by targeting VEGFA and Ang-2 in BC [98]. It has been revealed that angiopathologic role of Ang-2 could be mediated through activation of AT1R (Ang II type 1 receptor) and triggering VEGF/ RhoA-ROCK signaling [100]. AT1R could accelerate EMT, tumor growth, and neo-angiogenesis in BC [101]. ACE2 (angiotensin-converting enzyme 2) converts Ang II to Ang 1 and could interrupt BC angiogenesis by VEGFA downregulation [102].

MiR-199b-5p: MiR-199 family comprises two members: miR-199a and miR-199b. MiR-199b has been reported as downregulated miRNA in BC patients, and it can decrease proliferation and migration of BC cells [103]. Wu *et al.* also showed that TNBC lines exhibited low expression of miR-199b-5p, and its overexpression reduced their migration and invasion features [104]. According to study by Lin *et al.* miR-199b-5p can negatively regulate angiogenesis by targeting activin receptor-like kinase 1 (ALK1) [105]. ALK1 is serine/ threonine kinase receptor which is involved in TGFβ signaling pathway. The high expression of ALK1 was considered as the marker of metastasis in BC [106]. MiR-199b-5p directly targeted ALK1, and suppressed tube formation and migration of HUVEC. The knockdown of miR-199b-5p subsequently reduced the expression of downstream proteins including Smad1/5/8, Smad1, and inhibitor of DNA Binding 1, HLH Protein (Id1). Interestingly, miR-199b-5p effectively reduced growth and angiogenesis in breast tumors in mice [105].

MiR-200c: Jones *et al.* showed that the levels of miR-200c were decreased in the claudin-low BC cells due to the promoter hypermethylation. Over-expression of miR-200c inhibited cell proliferation and colony formation *in vitro*. MiR-200c also reduced tumor growth and tumor vasculature *in vivo*. FLT1 and VEGFC were identified as potential direct targets of miR-200c in this study and thus claudin-low BC cells may have a higher level of angiogenic markers [107]. Pecot *et al.* also found a significant inverse correlation between the levels of miR-200c and the pro-angiogenic cytokine, IL-8 and thus decreased miR-200c expression was associated with significantly worse overall survival in ovarian and basal-like breast cancer [108].

MiR-204: MiR-204 is an intronic miRNA located at chr 9. Downregulation of immune-related factors including Ccl20, CSF1, PDGFB and VEGFA by miR-204, in addition to the alteration of immune cells composition such as decreased myeloid-derived suppressor cell (MDSCs) and increased regulatory T cells population by miR-204 could limit metastatic potential of tumor cells therefore, miR-204 exerted immunomodulatory function in BC TME [109]. Since VEGFA could be suppressed by miR-204, it can be suggested besides immunomodulatory function, miR-204 has angioregulatory feature. Flores-Pérez *et al.* has disclosed that miR-204 could regulate tumor angiogenesis and repress tumor cells migration and proliferation [110]. They observed that co-culture of miR-204 transfected BC cell lines and HUVECs inhibited capillary tube formation. Moreover, angioreactor-based *in vivo* assay exhibited that BC cells transfected with miR-204 could inhibit angiogenesis. They showed that Ang1 and TGFβR2 are negatively regulated by miR-204. Suppression of Ang1 and TGFβR2 by miR-204 impaired capillary-like structures and branch points formation in HUVECs co-cultured with BC cell lines [110].

MiR-205: MiR-205 depletion leads to disruption of basement membrane (BM) and favors tumor cells metastasis and invasion [111]. BM loss of integrity in BC is associated with increased metastatic potential of tumor cells and facilitated access to blood vessels [112]. In addition, BM degradation is essential for angiogenesis. Yan-e Du *et al.* have shown that miR-205 inhibited Yes-associated protein (YAP1) expression that counteracted transformation of normal fibroblasts to CAFs [113]. These authors have demonstrated that activated CAFs secreted IL-11 and IL-15 following by STAT3 signaling activation in HUVECs that finally leads to tube formation and sprouting. Moreover, rescue experiments results using VEGF antagonist confirmed that miR-205 downregulation in CAFs mediates angiogenesis in VEGF independent manner both *in vitro* and *in vivo*. Altogether, it can be suggested that miR-205 has a critical role in angiogenesis through interaction of tumor cells and stroma.

MiR-206: Liang and co-authors by *in vivo* angiogenesis assay have indicated that miR-206 could abrogate angiogenesis in miR-206 administrated nude mice model of TNBC [114]. Their results showed that miR-206 overexpression correlated with VEGF downregulation in TNBC tissues and could suppress VEGF, SOX9, and MAPK expression in BC cells. Pro-angiogenic function of SOX9 has been shown in other types of cancer [115]. SOX9 overexpression is correlated with BC poor survival, Wnt signaling, and BC stem cell markers expression including ALDH and CD44 [116], [117]. RRM2-induced MAPK signaling participates in endothelial tube formation, migration, and invasion in BC [118]. Since SOX9 could be activated in downstream of MAPK signaling pathway, miR-206/MAPK/SOX9 would be involved in angiogenesis regulation of BC.

MiR-497: The ectopic expression of hypoxia-responsive tumor suppressor miR-497 by targeting VEGF and HIF-1α could reduce tube formation and MVD *in vitro* and *in vivo* under hypoxia condition*.* [119]. On the contrary, restored normoxia caused downregulation of VEGF and HIF-1α. Another study showed upregulated miR-497 by suppressing VEGFR2 caused activation of PI3K/AKT and MAPK signaling which could hamper angiogenesis [120].

MiR-519c: Cha *et al*. demonstrated that miR-519c targeted the HIF-1α, and subsequently reduced the tube formation in HUVECs. Similarly, the injection of cells with overexpressed miR-519c into mouse xerograph model significantly reduced HIF-1α and angiogenesis. [121].

MiR-542-3p: Injection of lentivirus containing miR-542-3p into orthotropic BC models reduced tumor angiogenesis and growth [122]. Anti-angiogenic activity of miR-542-3p rescued by Ang-2 overexpression moreover, *in vitro* angiogenesis assay confirmed these results [122]. Ang-2 /TIE2 signaling promotes BC metastasis [123] and stimulates angiogenic TIE2-expressing macrophages (TEM) function, In addition, Ang-2 inhibition or TIE2 antagonist leads to BC vasculature disruption [124], [125].

MiR-578 and miR-573: MiR-578 and miR-573 expression levels are associated with breast cancer 1 gene (BRCA1) gene status in BC, and play anti-angiogeneic role through regulating the angiogenic markers such as VEGF, focal adhesion kinase (FAK) and HIF-1 α [126]. FAK plays a fundamental role in tumor angiogenesis [127]. Steroid receptor coactivator (Src), FAK, and STAT3 signaling transduction contribute in angiogenesis of ER^+^ BC through paxillin (PXN) [128].

MiR-892b: Jiang *et al.* revealed that miR-892b levels were down-regulated in the BC cell lines due to the promoter hypermethylation and miR-892b was inversely correlated with the clinical stage and TNM classification in the BC patients. They further showed that tumor size and weight were higher in the miR-892b-silenced group. MiR-892b-silenced tumors also exhibited increased MVD and prominent lung metastasis. MiR-892b significantly reduced colony formation and the ability of BC cells to induce HUVEC tubule formation. MiR-892b over-expression decreased the expression levels of TRAF2, TAB3 and TAK1 in the BC cells and clinical specimens and thus miR-892b functions as a tumor-suppressive miRNA in BC by targeting multiple components in NF-κB cascade [129].

MiR-4306: Zhao and colleagues have demonstrated that miR-4306 expression could be negatively regulated by binding of ERα, progesterone receptor (PR), and human epidermal growth factor receptor 2 (HER2) to its promoter in TNBC cells [130]. They showed that TNBC-derived CM containing upregulated miR-4306 reduced lymphangiogenesis in human dermal lymphatic endothelial cells (HDLECs), and angiogenesis in HUVECs by SIX1 and VEGFA suppression respectively. Moreover, by *in vivo* angiogenesis examination, they confirmed *in vitro* results using chick chorioallantoic membrane (CAM) assay.

MiR-4500: The expression levels and the effects of miR-4500 in the BC angiogenesis was evaluated in the study conducted by Li *et al.* The levels of miR-4500 were reduced in the BC cells and RRM2 was identified as its target gene. MiR-4500 inhibited BC cell proliferation, migration and invasion by downregulating RRM2. The number of capillary-like tubes of ECs was significantly reduced as well as the levels of phosphorylated ERK, p38 and MEK in the miR-4500 mimic group. Thus, miR-4500 suppressed angiogenesis through MAPK signaling pathway *in vitro*. Further results showed that miR-4500 reduced the tumor growth rate and the tumor volume in tumor xenograft-induced nude mice via RRM2 downregulation [131]. The role of RRM2 in the BC angiogenesis was also proved in the study conducted by Zhuang *et al.* They showed that knockdown of RRM2 inhibited the migration and invasion of the BC cells and the release of VEGF in these cells [132].

### Pro-angiogenic miRNAs in BC

MiR-9: The results regarding the role of miR-9 in BC angiogenesis are controversial. Kim *et al*. have shown that miR-9 targets the transcripts of the genes involved in the activation of VEGF expression [133]. For instance, they showed that miR-9 is able to reduce the VEGFA expression in BC cell lines through targeting the transcripts of integrin subunit alpha 6 (ITGA6) gene encoding a subunit of α6β4 integrin complex [133]. It has been demonstrated that the α6β4 integrin can increase the translation of VEGF [134]. The comprehensive *in vitro* and *in vivo* study carried out by Ma *et al.* have revealed that miR-9 increases the VEGFA expression in BC expressing E-cadherin [135]. They showed that miR-9 targets the transcripts of *CDH1* gene encoding E-cadherin and enhances the activity and nuclear localization of β-catenin [135] which are both involved in tumor angiogenesis[136]. Finally, they indicated that overexpression of miR-9 increases the angiogenesis in breast tumors [135]. In addition, it has been reported that overexpression of long non-coding RNA (lncRNA) maternally expressed gene 3 (MEG3) in HUVEC cells reduces the tube formation in these cells and this inhibitory effect can be partially overcome by co-transfection of miR-9 [137]. Furthermore, the *in vitro* experiments have confirmed that lncRNA MEG3 directly binds to miR-9 and suppresses its activity [137]. Altogether, these data support the notion that miR-9 has a pro-angiogenic role in BC.

MiR-10b: The gene encoding miR-10b is located at the upstream of *homeobox protein Hox-D4* (*HOXD4*) gene [138]. Various studies have determined the significant role of miR-10b in angiogenesis using different *in vitro* and *in vivo* models [139]. High level of miR10b has been correlated with increased MVD in axillary lymph node‑negative BC patients [140]. Plummer *et al.* have revealed that ECs isolated from murine and human breast tumors have higher expression of miR-10b in comparison with normal ECs [141]. The overexpression of miR-10b has been proposed to increase the ability of tube formation in human microvascular endothelial cells through suppressing the expression of genes involved in anti-angiogenic pathways [142]. Transfection of HMEC-1 cells with specific vectors containing gene sequences encoding 3ʹ UTR region of HoxD10 mRNA and luciferase reporter have demonstrated that miRNA-10b targets HoxD10 mRNA and reduces the expression of the HoxD10 protein expression [142]. Moreover, treatment of mice harboring MDA-MB-231 xenografts with heparin decreases and increases miR-10b and HoxD10 expressions, respectively, which have potential role in anti-angiogenic activity of heparin [142]. Fms-related tyrosine kinase 1 (FLT1) and soluble FLT1 (sFLT1) have been demonstrated to be another target of miR-10 family [139]. It has been suggested that FLT1 can exert its anti-angiogenic effects through inhibiting VEGF and VEGFR2 interaction [143], [144]. Inhibition of miR-10 expression in HUVECs treated with low concentration of VEGF significantly reduces the phosphorylation of VEGFR2 which can ultimately impair angiogenesis in these cells [139].

MiR-20a: MiR-20a is an oncogenic miRNA belongs to miR-17/92 cluster encoded by the miR-17/92 cluster host gene (*MIR17HG*) [145]. Although, elevated expression of miR-20a has been shown in hormone receptor-negative as well as basal-like BCs [146] lower level of miR-20a has been determined in plasma of patients with invasive BC in comparison with normal samples [147]. Further analysis of miR-20a expression in breast tumors revealed that miR-20a is positively correlated with vessel size and VEGFA expression [146]. However, the ectopic expression of miR-20a in MCF-7 BC cells did not alter the expression level of genes involved in angiogenesis including VEGFA [146]. In addition, it has been shown that treating the ECs with CM of MDA-MB-231 transfected with anti-miR-20a can significantly reduce vascular area and size in these cells [146]. Taken together, it seems that miR-20a can regulate the breast tumor angiogenesis by collaboration with angiogenic factors through mostly unknown mechanisms.

MiR-21: MiR-21 is a well-known oncomiR in BC development. Bioluminescent monitoring of murine transgenic BC model harboring luciferase underlying VEGFR2 promoter revealed that tumor angiogenesis and growth could be attenuated following by intratumoral injection of anti-miR-21 which is associated to HIF-1α/VEGF/VEGFR2 signaling inactivation [148]. Furthermore, this study showed miR-21 suppression triggers apoptosis in BC cells and HUVECs by PTEN upregulation. This miRNA has also been demonstratd to suppress angiogenesis. However, BC exosomal miR-21 induced by anti-angiogenic omega-3 fatty acid named docosahexaenoic acid (DHA), was not able to reduce tube formation of endothelial cells [149]. Liu, Y *et al.* study demonstrated metadherin (MTDH), an oncogene in BC, could support angiogenesis through miR-21/ERK/VEGF/MMP2 axis [150]. They have shown suppression of PTEN expression by miR-21 thereupon upregulation of p-ERK1/2, VEGF and MMP2 is in a relationship with MTDH pro-angiogenic function. It has been shown that physical exercise and hormone therapy in BC orthotropic mouse model resulted in decreased tumor growth and angiogenesis through downregulation of miR-21, HIF-1α, ERα, VEGF and upregulation of miR-206, let-7a, programmed cell death protein 4 (PDCD-4), IL-10 [151] hence, miR-21 reduction is linked to anti-angiogenic response in BC. MiR-21 by inhibition of anti-angiogenic factors such as tissue inhibitor of metalloproteinase-3 (TIMP3) in tumor-infiltrating myeloid cells’ (TIMs) could promote metastatic tumor angiogenesis and proliferation through modulating the tumor microenvironment [152].

MiR-93: MiR-93 is an intronic miRNA on chr 7 which belong to the miR-106b-25 cluster. Ectopic expression of miR-93 in TNBC cell line by suppression of WNK1 (WNK lysine deficient protein kinase 1) attenuated invasion and migration of tumor cells [153]. WNK1 is a pro-angiogenic factors that leads to tumor progression [154]. MiR-25/93 are hypoxia-responsive miRNAs that could repress NCOA3 and impair cyclic-GMP-AMP synthase (cGAS)-mediated anti-tumor immunity in BC [155]. Therefore, hypoxia-regulated miRNAs may contribute to tumor angiogenesis besides immunosuppression. MiR-93 regulates tumor angiogenesis by suppressing various targets including VEGF, IL-8, epithelial protein lost in neoplasm (EPLIN), large tumor suppressor, homology 2 (LATS2) and integrin-β8 [156]. High expression of miR-93 is correlated with elevated vascular density in TNBC tissue, moreover, EPLIN inhibition by miR-93 could enhance HUVECs tube formation, sprouting, proliferation and migration [156]. Low level of EPLIN in BC is related to increased cell motility, angiogenesis, and poor clinical outcome [157], [158]. Fang *et al*. have shown tumor xenograft arising from miR-93 transfected BC cell line showed higher vessel density and lung metastatic potential compared to control vector-transfected tumors [159]. In addition, they observed that miR-93 by targeting LATS2 supported tumor angiogenesis and invasion. MiR-93 could reduce integrin-β8 which might confer intercellular interaction between tumor cells and ECs and angiogenesis facilitation [159], [160].

MiR-182: Oncogenic miR-182 is a member of the miR*-*183*/*182*/*96 cluster located on chr 7q32 region. MiR-182-overexpressing is associated with upregulation of HIF-1α and VEGFA by targeting F-box and 7 WD repeat domain-containing 7 (FBXW7), as a result promotes angiogenesis in BC [161]. Tumor suppressor FBXW7 is one of the crucial components of ubiquitin ligase called Skp1- Cullin1-F-box (SCF) complex that controls the degradation of a number of oncoproteins including cyclin E, Notch, c-myc, and HIF-α [162].

MiR-210: In the study by Jung *et al.* the high expression of HIF-1α and miR-210 was observed in the exosome derived from mouse BC cells under hypoxic condition. The transfection of miR-210 containing exosomes into HUVEC cells effectively reduced the expression of Ephrin-A3 and PTP1B and promote VEGF- mediated angiogenesis [163].

MiR-467: Bhattacharyya *et al.* identified miR-467 as a negative regulator of (TSP-1) that was induced after glucose stimulation in the BC cells. The miR-467 mimic increased the number of BC cells in the matrigel plugs in mice, suggesting the proangiogenic activity of miR-467 *in vivo*. However, miR-467 failed to increase angiogenesis in the TSP1-knockout mice. Furthermore, it was shown that the level of miR-467 significantly increased in the BC tumors and its level tended to correlate with tumor mass in STZ-treated hyperglycemic mice. Similar results were also seen in the hyperglycemic Lepr^db/db^ mice, suggesting that hyperglycemia-induced miR-467 increased the tumor size and angiogenesis [164]. These results were further proved in the study conducted by Krukovets *et al*. They injected mouse BC cell lines (EMT6, Ac711, MMTV-Wnt-1) and human BC cell line (MDA-MB-231) into the mammary fat pad of Lepr^db/db^ and NU/J mice, respectively. They evaluated tumor growth and angiogenesis markers (CD31, laminin-1, α-actin) in these mouse models. They revealed that miR-467 antagonist inhibited tumor growth and decreased angiogenesis markers in the mouse models [164]. These studies demonstrated that hyperglycemia induced angiogenesis via miR-467 induction.

MiR-562 and miR26b*: MiR-562 and miR26b* are tumor suppressor miRNAs located on human chromosomes 2q37.1 and 2q35 respectively [165]. MiR562 and miR26b* by direct suppressing the NF-κB subunit RELA (p65) and NF-κB1 (p105) are associated with angiogenesis in BC [166]. The NF-κB signaling is correlated with several pathways including PI3K /AKT signaling pathway [167]. PI3K/AKT signaling is involved in HIF-1α and VEGF induction and plays a critical role in BC angiogenesis [168], [169]. Hence, miR-562 and miR26b* induce BC angiogenesis through NF-κB /PI3K /AKT/HIF-1α/VEGF axis.

MiR-526b/655: MiR-526b/655 are significantly upregulated in BC and associated with poor prognosis [170]. The *in vitro* studies indicated that miR-526b/655 induced by COX2, an inflammatory enzyme which is upregulated in BC [171], [172]. Hunter *et al.* investigated the role of miR-526b and miR-655 in BC angiogenesis. They indicated that miR-526b and miR-655 transfection resulted in upregulation of angiogenic proteins including VEGFC, VEGFD, COX2, and LIVE1. The VEGFC is the marker of angiogenesis and VEGFD and LYVE1 are the marker of lymphangiogenesis. They also observed the upregulation of VEGFR1 in the cell lines with both miRNAs. Interestingly, the media containing miR-526b and-655 caused tube formation in HUVEC cells [173].

## MiRNAs in BC vasculogenic mimicry

**Fig. 4:**
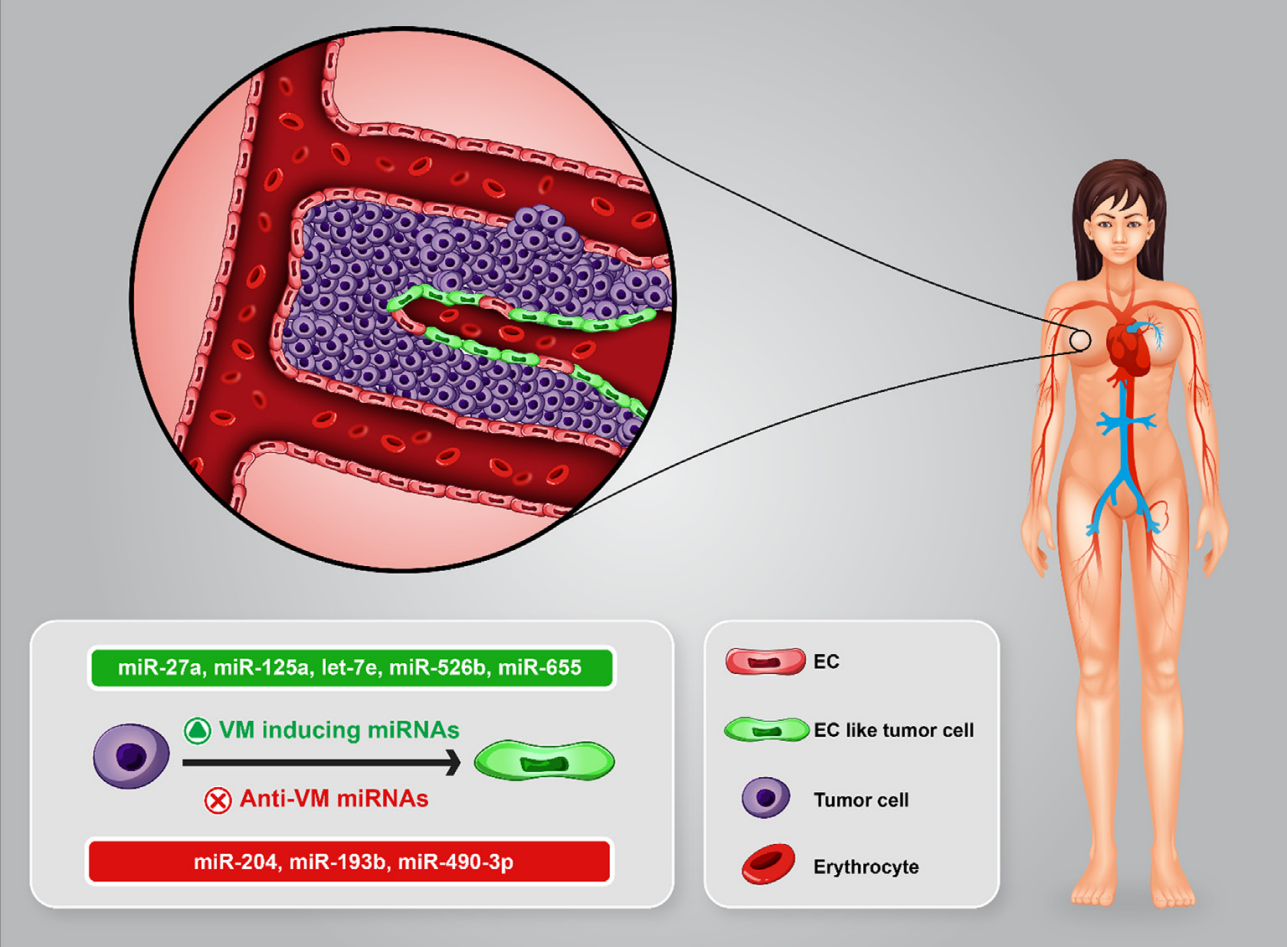
Angioregulatory miRNAs in VM. BC cells transdifferentiated to EC-like tumor cell. VM is the ability of forming de novo vascular networks in aggressive tumor cells, moreover vessel sprouting together with VM can support tumor growth. Angioregulatory miRNAs can promote or suppress this process in BC. EC, endothelial cells; VM, vasculogenic mimicry.

**Fig. 5:**
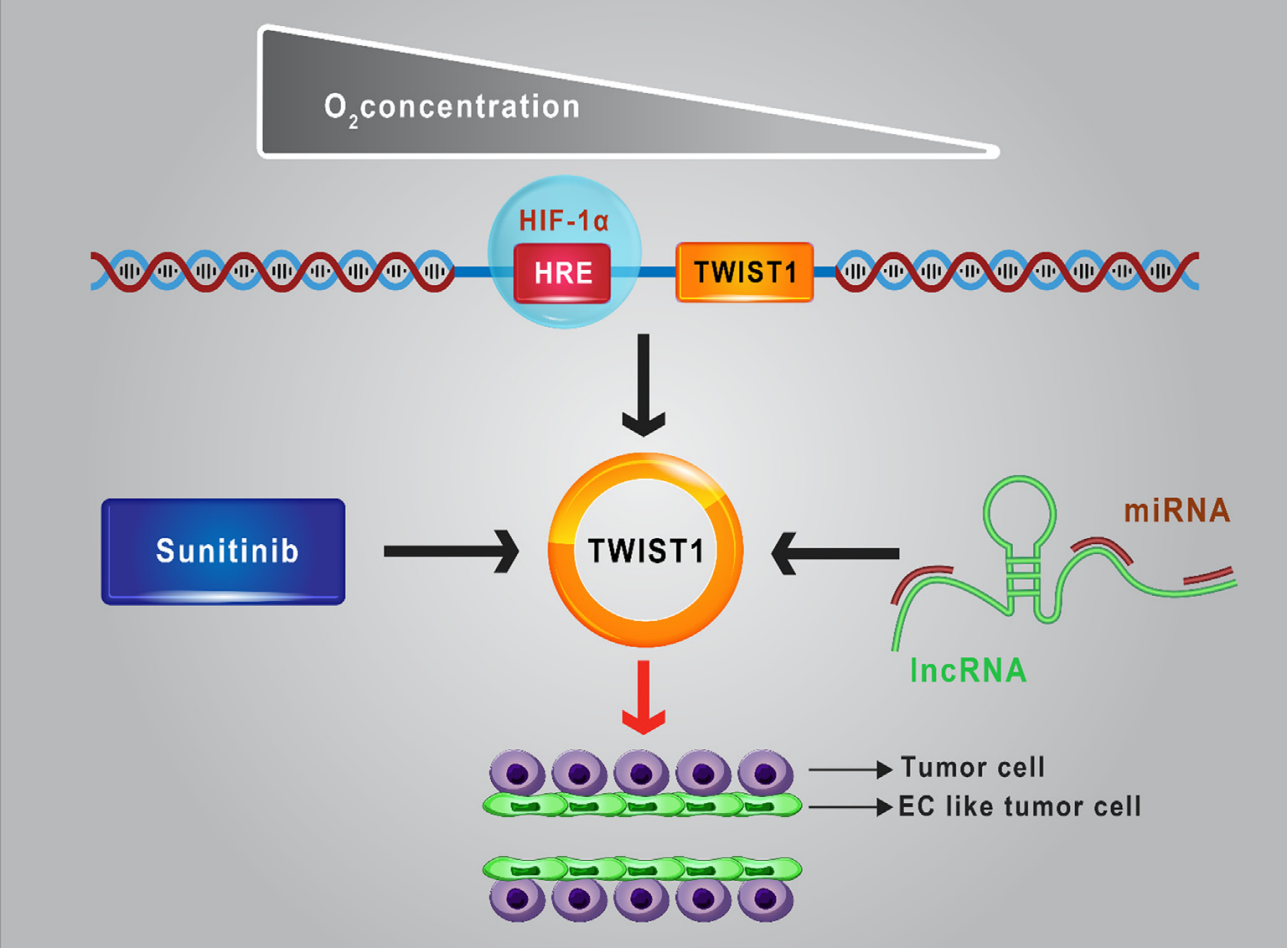
HIF-1α/TWIST1/miRNA axis in VM. HIF-1α by binding to HRE can induce TWIST1 under hypoxia condition. Regulating TWIST1 expression by angioregulatory miRNA and Sunitinib can trigger VM. This phenomenon may be involved in anti-angiogenic therapy resistance. HIF-1α, hypoxia inducible factor 1 subunit alpha; HRE, hypoxia-response element; EC, endothelial cells; LncRNA, long non-coding RNA.

Vasculogenic mimicry (VM) is the ability to form de novo vascular networks in aggressive tumor cells [61]. VM involvs in formation of tubular and patterned matrix by tumor cells results in mimicking ECs functions, and thereby is strongly associated with metastatic behavior, anti-angiogenic therapy resistance, and poor clinical outcome in BC [174]. ALDH^+^ and CD133^+^ breast cancer stem-like cells-lined blood vessels play a critical role in VM and aggressive tumor growth [175], [176]. Indeed, not only blood vessels could be formed by the requirement of ECs, but also could be generated from tumor-initiating cells. Mesenchymal BC cells secreted VM-promoting factors such as fibronectin 1 (FN1), serine protease inhibitor (serpin) family E member 2 (SERPINE2) under nutrient deprivation that could be induced VM through paracrine signaling. MiRNAs repudiated VM in BC by repression of these factors and their receptors that could be reversed by zinc finger E-box binding homeobox 1 (ZEB1) overexpression [177]. Therefore, VM can be regulated by angioregulatory miRNAs in BC (Fig. 4). MiR-27a could regulate BC stem-like cells differentiation into ECs and induce VEGF-mediated angiogenesis and metastasis [178]. Expression of specificity protein 1 (Sp1), VEGF, and VEGFR2 were increased following by zinc finger and bTb domain containing 10 (ZBTB10) suppression via miR-27a/Runx1 axis [178]. Sp1 and HIF-1α overexpression after Bevacizumab treatment is involved in anti-angiogenic resistance in tumor cells [179]. Therefore, Sp1 downregulation due to miR-27a suppression might be implicated in attenuating BC resistance to anti-angiogenic therapy. MiR-193b is another mediator of VM, can target dimethylarginine dimethylaminohydrolase (DDAH1). High expression of dimethylarginine dimethylaminohydrolase (DDAH1) is related to de novo microvascular channels organization by TNBC cell line, in addition, DDAH1 expression suppression by miR-193b could impede VM and VEGFA expression in BC [180]. Wei Tao *et al*. have shown miR-490-3p by silencing Twist1 could regulate VM in the TNBC cell line [181]. They have demonstrated that lncRNA TP73-AS1 as a negative regulator of miR-490-3p by binding to this tumor suppressor miRNA could abrogate its function in VM suppression. Twist1 expression due to sunitinib treatment could induce CD133^+^ BC progression and VM under hypoxia condition [182]. HIF-1α binds to the hypoxia response element (HRE) in Twist1 promoter confer EMT phenotype and CSC-derived progression of invasive tumors [183], [184] (Fig. 5). It has been concluded that uncovering the miRNA/HIF-1α/Twist1 regulatory axis in BC exerts an important role in VM. Two miRNAs, miR-125a and Let-7e have been shown to inhibit VM in BC cells by targeting IL-6 signaling pathway. Park *et al*. have evaluated the relation between VM and chemotherapy resistance which is triggered through intercellular communication between ECs and TNBC cell line (MDA-MB-231). They found that cisplatin containing media promoted tube formation through inducing IL-6, and miR-125a/ let-7e overexpression reversed this effect in TNBC cells [185].

Salinas-Vera and co-authors have shown miR-204 could suppress VM in the TNBC cell line via targeting HIF-1α [186]. They have implicated that phosphorylation status of multiple proteins including FAK, c-SRC, Elk1, VEGFR1/2, p-38α MAPK, AKT, PI3K-α could be changed directly or indirectly under hypoxia in BC due to miR-204 transfection into BC cells. Hence, it could be suggested that BC-mediated VM formation is intensively modulated by various signaling pathways such as PI3K/AKT, MAPK, VEGF and FAK/SRC which are also common to EC-dependent angiogenesis. Gervin *et al*. have recently investigated the relation between hypoxia and expression of miR-526b/655, and showed that hypoxia significantly increased angiogenesis markers including HIF-1α, COX-2, EP4, NF-KB1 and VEGFA in the cells expressing high level of miRNAs Hypoxia promoted neovascularization in the miR-526b/655-expressing cells. Notably, VM was only observed in MCF7-miR526b and MCF7- miR655 but not in MCF7 cells [170].

## Angioregulatory miRNAs modulation by anti-angiogenic treatment and translational implications of miRNAs-based therapeutics in BC

Using combinational therapies such as targeted therapies, chemotherapies, photodynamic therapies and radiotherapies with miRNAs-based therapies might be more beneficial in cancer treatment [252]. The significant roles of miRNAs dysregulation in BC angiogenesis would prompt researchers to develop anti-angiogenic compounds to modulate their expression. Anti-tumor miRNAs-based therapeutics strategies can be relayed on miRNAs mimics and antagonist to target angiogenic-related factors. However, these approaches are still in infancy.

Molecular heterogenicity in BC causes different responses to anti-angiogenic targeted therapy. For exmple, alteration in expression of miRNAs can predict responsive vs non-responsive patients. Phase III clinical trial in BC patients treated with Bevacizumab showed that low expression of miR-20a-5p as a the predictive biomarker in the tumoral tissues was correlated with better clinical outcome [253]. Phase II clinical trial in BC responder to Bevacizumab showed increased tumor suppressor miRNAs and decreased onco-miRNAs which among them miR-4465 has the most significant correlation with tumor growth suppression [254]. Although, due to no significant effect on overal survival, BC treatment with Bevacizumab has been withdrawn, however identification of miRNAs-based prognostic and predictive biomarkers might improve clinical benefits in BC patients. Circulating miRNAs have been emerged as a novel and accurate biomarkers that could be achived with less-invasive and easily accessible approcahes in numorus types of cancer. High speceficity and sensitiviy of miRNAs highlighted these class of molecules as the favorite tumor biomarkers. Because of cancer molecular heterogenecity, presonalized multi-miRNAs based panel would be more precise biomarkers. However, reproducibility and methodological issues are still serious obstacles in this era.

**Fig. 6:**
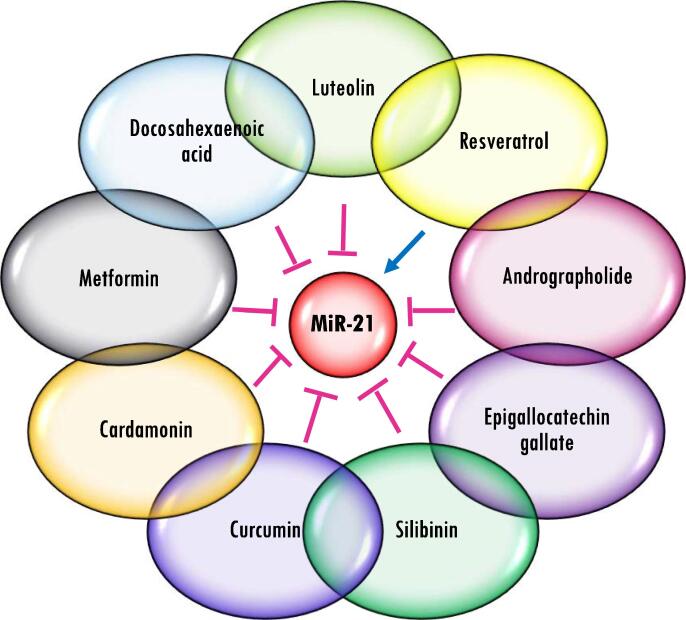
MiR-21 is a key angioregulatory miRNA in anti-angiogeneic therapy response. MiR-21 is mostly reduced in response to anti-angiogenic effect of phytochemical drugs. Although, Resveratrol activates miR-21 expression, it can inhbite others angio-prmoting miRNAs in BC.

Phytochemical drugs including Resveratrol, Curcumin, Genistein, Cardamonin, etc. could modulate angioregulatory miRNAs (especillay miR-21) (Fig. 6) expression which are involved in VEGF/HIF-1α axis, glycolytic pathways and reactive oxygen species (ROS) signaling in BC [255]. Cyanidin‐3‐glucoside anti-oxidant and anti-inflammatory drug, could decrease STAT3 expression through miR-124 overexpression that would eventually lead to reduced VEGF expression and diminished angiogenesis in BC [256]. Mu *et al*. demonstrated Glabridin a flavonoid compound, can upregulate miR-148a in BC cells [257]. They further showed Glabridin-induced miR-148a negatively regulated Wnt/β-catenin signaling which led to VEGF downregulation and angiogenesis suppression. Docosahexaenoic acid (DHA) treatment of BC cell lines under cobalt chloride‐induced hypoxia caused downregulation of onco-miRNAs (miR-21 and miR-382), on the contrary tumor suppressors miRNAs (miR-199 and miR-101) upregulated in BC cells and their exosomes. Moreover, pro-angiogenic content of BC cells and their corresponding exosomes including HIF1-α, TGF-β, SOX2, Snail1, Snail2 and VEGFR were decreased following DHA treatment. [258]. Chick embryo CAM, yolk sac membrane (YSM) assay and *ex vivo* rat aortic rings assay, in addition to *in vitro* angiogenesis assay showed anti-angiogenic effects of Andrographolide (labdane diterpenoid) in BC xenografts and HUVECs. This inhibitory effect could be mediated by miR-21-5p downregulation and then upregulation of its target, tissue inhibitor of metalloproteinase-3 (TIMP3) [259]. Therefore, it can be suggested that angioregulatory miRNAs can be used as the predictive biomarkers of response for phytochemical drugs.

**Fig. 7:**
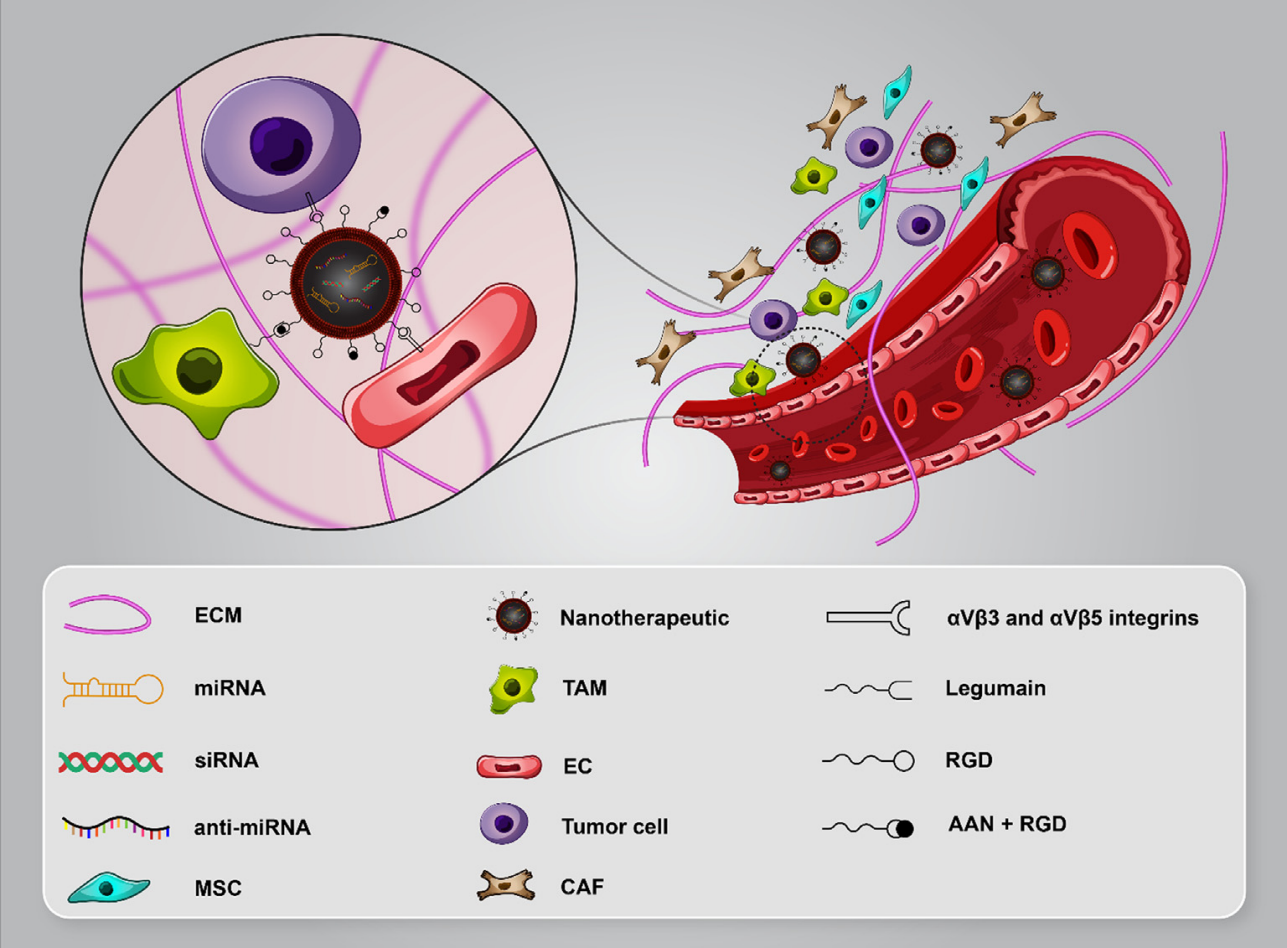
Nanotherapeutics containing miRNAs or miRNA antagonists in BC therapy. Specefic delivery of nanotherapeutics such as RGD-conjugated liposome and AAN-RGD liposomes can inhibit tumor angiogenesis by simultanously targeting TME cells.

The major challenges in anti-angiogenic therapeutics including resistance and toxicity need to be addressed. The liposomal-encapsulated miR-34a, MRX34, has been proved to induce serious immunologic side effects in phase I clinical trial (ClinicalTrials.gov Identifier: NCT01829971)) [260]. Employing modified nanocarriers such as cyclic arginine-glycine-aspartic acid (RGD) peptide conjugated nanoparticles containing miRNAs or anti-miRNAs could boost angiogenesis targeted therapy by binding to αvβ3 and αvβ5 integrin on tumor cells and tumor-associated endothelial cells (TEC) efficiently and specifically [187], [261]. Lipocalin 2 small interfering RNA (siRNA) encapsulating liposome by binding to intercellular adhesion molecule-1 (ICAM-1) reduced VEGFA expression and angiogenesis in TNBC [262]. Multi-targeted therapy by adding alanine-alanine-asparagine (AAN) to RGD for simultaneously targeting ECs and tumor associated macrophages (TAM) showed significant synergistic anti-tumoral effect in BC [263]. TAM induced CCL18 secretion and promoted angiogenesis in BC [264]. Hence, specific delivery platform of anti-angiogenic factors into cancer cells and tumor microenvironment would be a prominent approach in BC therapy (Fig. 7). Anti-angiogenic resistance mechanisms including tumor dormancy, vascular mimicry, vessel co-option, and alternative angiogenic pathways activation such as delta-like ligand-4 (DLL4)-Notch signaling, upregulation and polymorphisms of growth factors cause poor efficacy of anti-angiogenic therapeutics that can be mediated by aberrantly expressed miRNAs. Although, miRNAs may reverse resistance to anti-angiogenic therapy [265]. Altered miRNAs in dormant tumor cells could impaired angiogenesis by repressing pro-angiogenic factors like HIF-1α, and TIMP3 [266]. Exosomal miRNAs are involved in communication between MSC and BC which could mediate tumor dormancy [267]. It seems anti-angiogenic miRNAs delivery to tumor cells by targeting endogenous angiogenesis inhibitors may induce dormant BC cells which constitutes low proliferation and invasive potential, hence, tumor dormancy is a double-edged sword in cancer treatment. Altogether, therapeutic outcome of all these approaches are needed to be further validated in experimental setting.

## Conclusion and future perspectives

Successful targeting of tumor angiogenesis is still a missing link in the treatment of BC due to the low effectiveness of anti-angiogenic therapies in this cancer. The identification of new molecular aspects in the regulation of angiogenesis, in addition to a better understanding of tumor growth mechanisms, will help design effective therapeutics. A wide range of genes involved in angiogenesis moreover, response to anti-angiogenic therapeutics are controlled by a miRNAs, so the identification of interaction networks of miRNAs-targets can be applicable in determining anti-angiogeneic therapy and new biomarkers in BC. Conducting clinical trials to specifically deliver nano-therapeutics containing miRNAs or anti-miRNAs into the tumor cell and/or tumor microenvironment cells may be effective in developing a new generation of drugs based on angioregulatory miRNAs. On the other hand, neoadjuvant phytochemical drugs may improve the survival of BC patients by modulating the expression of angioregulatory miRNAs. Altoghther, we clarify the inhibitory and stimulatory mechanisms of angioregulatory miRNAs in BC-related angiogenesis pathways which can be a potential anti-angiogenic approaches in BC therapies.

Compliance with Ethics Requirement

Our article does not contain any studies with human or animal subjects*.*

## CRediT authorship contribution statement

**Mohammad Hasan Soheilifar:** Conceptualization, Writing - original draft. **Nastaran Masoudi-Khoram:** Writing - review & editing. **Soheil Madadi:** Writing - review & editing. **Sima Nobari:** Writing - review. **Hamid Maadi:** Writing - review. **Hoda Keshmiri Neghab:** . **Razieh Amini:** . **Mahboubeh Pishnamazi:** Conceptualization, Writing.

## Declaration of Competing Interest

The author declare that there is no conflict of interest.
